# Let’s talk about Secs: Sec61, Sec62 and Sec63 in signal transduction, oncology and personalized medicine

**DOI:** 10.1038/sigtrans.2017.2

**Published:** 2017-04-28

**Authors:** Maximilian Linxweiler, Bernhard Schick, Richard Zimmermann

**Affiliations:** 1 Department of Otorhinolaryngology, Head and Neck Surgery; Saarland University Medical Center, Homburg, Germany; 2 Institute of Medical Biochemistry and Molecular Biology, Saarland University, Homburg, Germany

## Abstract

The heterotrimeric Sec61 complex and the dimeric Sec62/Sec63 complex are located in the membrane of the human endoplasmic reticulum (ER) and play a central role in translocation of nascent and newly synthesized precursor polypeptides into the ER. This process involves targeting of the precursors to the membrane and opening of the polypeptide conducting Sec61 channel for translocation. Apart from this central role in the intracellular transport of polypeptides, several studies of the last decade uncovered additional functions of Sec proteins in intracellular signaling: Sec62 can induce ER-phagy in the process of recovery of cells from ER stress and the Sec61 channel can also act as a passive ER calcium leak channel. Furthermore, mutations, amplifications and an overexpression of the *SEC* genes were linked to various diseases including kidney and liver diseases, diabetes and human cancer. Studies of the last decade could not only elucidate the functional role of Sec proteins in the pathogenesis of these diseases, but also demonstrate a relevance of Sec62 as a prognostic and predictive biomarker in head and neck cancer, prostate and lung cancer including a basis for new therapeutic strategies. In this article, we review the current understanding of protein transport across the ER membrane as central function of Sec proteins and further focus on recent studies that gave first insights into the functional role and therapeutic relevance of Sec61, Sec62 and Sec63 in human diseases.

## Protein transport into and across the ER membrane

**Figure 1:**
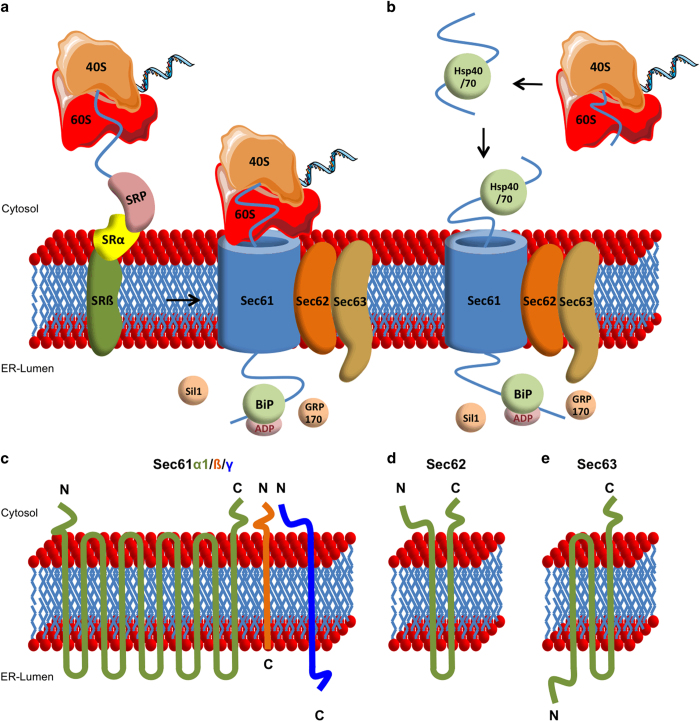
Protein transport across the endoplasmic reticulum membrane. Mechanism of (**a**) co-translational and (**b**) posttranslational transport of precursor proteins through the Sec61 channel. (**c**) Topological domains of Sec61α1/ß/γ, (**d**) Sec62 and (**e**) Sec63. We note that (i) Sec63 interacts with Sec62 involving a cluster of negatively charged amino-acid residues near the C terminus of Sec63 and positively charged cluster in the N-terminal domain of Sec62,^43^ (ii) Sec62 interacts with the N-terminal domain of Sec61α via its C-terminal domain,^68^ (iii) BiP can bind to ER luminal loop 7 of Sec61 α via its substrate-binding domain and mediated by the ATPase domain of BiP and the J-domain in the ER luminal loop of Sec63,^53^ (iv) Ca^2+^-CaM can bind to an IQ motif in the N-terminal domain of Sec61α^64^ and (v) LC3 can bind to a LIR motif in the C-terminal domain of Sec62.^71^ 40S, 40S ribosome subunit; 60S, 60S ribosome subunit; SR, heterodimeric SRP receptor; SRP, signal recognition particle.

The transport of precursor proteins into and across the endoplasmic reticulum (ER) membrane represents a highly conserved process in eukaryotic cells and is essential for the biogenesis of many transmembrane and most secretory proteins.^1–3^ Basically, this process can be divided into three major steps as follows: (i) the targeting of nascent and newly synthesized precursor polypeptides to the ER membrane; (ii) the insertion of the protein into the polypeptide conducting channel; and (iii) the lateral release of the transmembrane protein from the channel into the phospholipid bilayer or the completion of translocation into the ER lumen. As there are some mechanistic differences depending on the precursor protein being translocated during or after its synthesis at the ribosome, one can distinguish between the cotranslational^4,5^ (Figure 1a) and the posttranslational transport mechanism^6,7^ (Figure 1b). During co-translational transport, the ribonucleo-complex signal recognition particle (SRP)^8^ binds to a hydrophobic signal sequence located at or near the N terminus of the nascent precursor polypeptide and to the ribosome.^9^ Subsequently, the SRP receptor guides the ribosome nascent chain complex to the polypeptide conducting channel Sec61.^10^ Following GTP hydrolysis, SRP dissociates from the ribosome and the SRP receptor^11–13^ inducing a resumption of protein synthesis and the nascent polypeptide chain inserts into the Sec61 channel. Subsequently, membrane proteins diffuse laterally from the Sec61 complex into the bilayer. Alternatively, ER luminal chaperone proteins such as BiP/Grp78 can function as ‘molecular ratchets’ and guarantee the unidirectional transport of the nascent protein through the Sec61 channel into the ER lumen.^14–16^ To facilitate an interaction between these chaperones and the precursor polypeptides in transit, J domains of ER transmembrane proteins such as Sec63 mediate their direct interaction.^17–23^ As the activity of ER luminal BiP depends on ATP hydrolysis, the nucleotide-exchange factors Sil1 and GRP170 guarantee a replacement of ADP with ATP.^24^ During or after the precursor protein translocation is completed, the signal sequence is cleaved off by the signal peptidase complex,^25^ which is followed by folding of the translocated protein and covalent modifications such as N-glycosylation.^26^

The posttranslational transport is characterized by some crucial differences compared with the above-described co-translational transport mechanism: The precursor proteins are fully synthesized at free ribosomes because they bear a signal sequence of relatively low hydrophobicity (in yeast), or are simply too short (in mammals) to efficiently and productively interact with SRP at the ribosome, which leads to a completion of translation in the cytosol.^27,28^ To maintain a protein structure compatible with translocation across the ER membrane, cytosolic Hsp40 and Hsp70 chaperones prevent extensive protein folding at this stage and keep the signal sequence free for interaction with receptors at the ER surface.^29–31^ Depending on structural characteristics of the synthesized protein, for example, the chain length and the extent of folding, SRP as well as Sec62 can be required for an efficient targeting to the Sec61 translocon.^23,32–34^ The subsequent steps of protein transport are comparable with the co-translational transport. Figures 1a and b give an overview of the co- and posttranslational mechanism of protein transport into and across the ER membrane.

For both the co- and posttranslational transport, the protein translocation machinery as core element is composed of the ER transmembrane proteins Sec61, Sec62 and Sec63 (^ref. 35^) with Sec61 being composed of the three subunits Sec61α, Sec61ß and Sec61γ.^36–41^ These Sec proteins form oligomers with direct interactions of Sec62 and Sec63 to each other as well as to the Sec61 channel.^42,43^ However, only Sec61 and Sec62 but not Sec63 can directly interact with the ribosome.^43^ The topological domains of Sec61, Sec62 and Sec63 are shown in Figures 1c–e.

Sec61 as central element of the protein translocation machinery forms a protein conducting channel with an aqueous central pore.^44^ Precursor polypeptides can either completely cross the ER membrane through the Sec61 channel or exit laterally into the lipid phase of the membrane if the protein contains hydrophobic transmembrane domains.^45^ This evolutionarily conserved heterotrimeric mammalian ortholog to the bacterial protein SecY was characterized in detail in its structure and function over the past years.^10,36–41,46^

After its first functional and topological description in *Saccharomyces cerevisiae* in the late 1980s,^47–49^ a homolog to yeast Sec62p in mammals was identified in 1997.^35,42,50^ In mammalian cells, Sec62 interacts with Sec61 as well as Sec63 and, contrary to its yeast ortholog, harbors two conserved peptide domains at its cytosolic N terminus allowing a binding to the ribosome.^43^ The detailed function of Sec62 in the protein translocation process still remains uncertain though several studies of the past decade indicated a role of Sec62 in posttranslational transport: Lakkaraju *et al.* found that posttranslationally transported precursor proteins comprising ⩽100 amino acids strongly and precursor proteins comprising 120–160 amino acids partially depend on Sec62 for efficient translocation.^33^ In a study by Lang *et al.*, *SEC62* silencing led to a reduced ability for posttranslational import of small presecretory proteins without any impairment of co-translational transport or posttranslational membrane insertion of tail-anchored proteins.^23^ These findings are consistent with comparable observations in yeast,^51,52^ where Sec62p together with Sec61p, Sec63p, Sec71p and Sec72p forms the so-called ‘posttranslational translocon’. Against this background, the question about the functional relevance of the ribosome-binding site of Sec62 remains unanswered. Eventually, further studies are needed to elucidate the detailed function of Sec62 in the protein translocation process.

The Sec63 protein consists of three transmembrane domains with the ER luminal loop harboring a J-domain that allows an interaction with chaperones—such as BiP—to facilitate the unidirectional translocation of precursor proteins through the Sec61 translocation pore. Moreover, Lang *et al.* reported a precursor-specific role of Sec63, in cooperation with BiP, in the early phase of co-translational protein transport with proteins as pERj3, PrP and ppcecA being dependent on Sec63 for efficient initial insertion into the Sec61 channel.^23,53^ However, Görlich and Rapoport showed that neither Sec63 nor BiP is required for an efficient translocation of several other precursor proteins as preprolactin and VSV G protein in an *in vitro* protein transport model.^10^ Hence, the mechanism of how Sec63 acts in transport as well as its substrate specificity remains elusive. The testing of further potential substrates will be required to clarify which subset of proteins is dependent on Sec63 for efficient transport.

In addition to Sec-dependent transport of precursors with N-terminal signal peptides or internal transmembrane helices serving as signal sequences, there is a posttranslational mechanism for tail-anchored membrane proteins with C-terminal tail anchors.^3^ This mechanism involves cytosolic and membrane proteins for targeting and membrane integration, the so-called transmembrane recognition complex (reviewed in Borgese and Fasana^3^).

## Role of sec proteins in cellular Ca^2+^ homeostasis and autophagy

**Figure 2:**
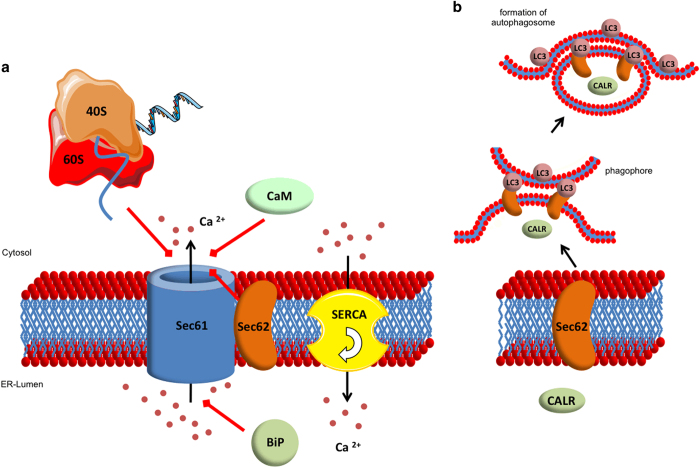
Regulation of Ca^2+^ homeostasis at the endoplasmic reticulum membrane and Sec62-mediated autophagy. (**a**) Regulation of Ca2+ efflux through the Sec61 channel. (**b**) Sec62-mediated autophagy. The red arrows in **a** indicate inhibitory effects on the passive Ca^2+^ efflux through the Sec61 channel. 40S, 40S ribosome subunit; 60S, 60S ribosome subunit; CaM, calmodulin; CALR, calreticulin; LC3, 1A/1B-light chain 3; SERCA, sarcoplasmic/endoplasmic reticulum Ca^2+^-ATPase.

An additional function for Sec61 apart from protein translocation was suggested by several studies, indicating that Sec61 could also serve as a Ca^2+^ channel that allows a passive efflux of Ca^2+^ ions from the ER lumen—the largest intracellular Ca^2+^ store—to the cytosol.^37,54–61^ Thus, Sec61 counteracts the active import of Ca^2+^ into the ER lumen through the sarcoplasmic/ER Ca^2+^ ATPase.^62^ In fact, Lang *et al.*^63^ were the first who could directly measure this Ca^2+^ flow through the open Sec61 channel in planar lipid bilayer experiments and link it to the Sec61 complex at the cellular level by siRNA-mediated knockdown experiments in combination with live-cell calcium imaging.^64^ As the cytosolic Ca^2+^ level crucially influences essential cellular processes as cell migration^65^ and apoptosis^66^—processes that both are seriously disturbed by an uncontrolled Ca^2+^ efflux from the ER^67^—it was suggested that the passive Ca^2+^ efflux through the Sec61 channel is regulated by diversified mechanisms: as first mode of regulation, Erdmann *et al.*^64^ could show that cytosolic calmodulin (CaM) can efficiently bind to the cytosolic site of Sec61 in a Ca^2+^-dependent manner, thus limiting Ca^2+^ efflux from the ER lumen. In addition, the chaperone BiP was shown to decrease the Sec61-mediated Ca^2+^ efflux too, by binding to the loop 7 of Sec61α from the ER luminal site.^53^ As a third regulatory mechanism, Linxweiler *et al.*^68^ showed that *SEC62* silencing increases the Ca^2+^ efflux from the ER, suggesting that the Sec62 protein also contributes to limit the Sec61-mediated Ca^2+^ flow (Figure 2a). As a point mutation (D308A) in a putative C-terminal EF hand domain of Sec62 failed to rescue the effect of Sec62 depletion on Ca^2+^ efflux contrary to a transfection of the cells with a wild-type plasmid,^68^ this regulatory effect is probably mediated by the C-terminal EF hand motif of Sec62. According to our working model, Ca^2+^ efflux leads to Ca^2+^ binding to the EF hands of Sec62 and CaM, conformational changes in these two proteins, and subsequent dissociation of Ca^2+^-Sec62 from the N terminus of Sec61α and simultaneous binding of Ca^2+^-CaM to an IQ motif in this N-terminal domain. Apart from that, Crowley *et al.*^69^ hypothesized based on transport experiments with fluorescently labeled translocation substrates and ER luminal iodide ions for collisional quenching that the Sec61 channel is impermeable to ions during the translocation of a nascent chain with the ribosome bound to Sec61. To what extent the Sec61-mediated Ca^2+^ efflux from the ER lumen and its regulatory mechanisms contribute to the global cellular Ca^2+^ homeostasis and how this is orchestrated with the other mechanisms of intracellular calcium signaling^70^ are important questions that have to be addressed by future studies.

An additional function beyond protein translocation and calcium homeostasis was recently found for the Sec62 protein as well. Fumagalli *et al.*^71^ showed that Sec62 plays a crucial role in the recovery of eukaryotic cells from conditions of ER stress. The term ER stress describes conditions under which the homeostasis of protein synthesis, folding and transport at the ER is disturbed due to the perturbation of ER environment.^70^ Depending on the severity of stress, the cell can either initiate compensatory mechanisms that are entirely referred to as unfolded protein response or undergo programmed cell death,^70^ which involves—possibly Sec61 channel mediated—Ca^2+^ efflux from the ER. During unfolded protein response, ribosomal synthesis of the majority of proteins is blocked, the expression level of several ER luminal chaperones, such as BiP and Herp, is markedly increased to facilitate a correct folding of ER luminal polypeptides and, in the case of defective repair, misfolded proteins are degraded via a mechanism called the ER-associated protein degradation.^72,73^ If the cell can finally cope with ER stress conditions, the expanded ER itself as well as the high amount of ER luminal chaperones have to be downsized to a physiological level again. Therefore, small vesicles bearing ER luminal and membrane chaperones are separated from the ER membrane, fuse with phagophores to build autophagosomes and finally are delivered to lysosomes for degradation—a process called autophagy (Figure 2b).^74^ In this context, Sec62 was shown to bear a LIR motif at its C terminus functioning as a receptor for phagophore-bound LC3 on recovery from ER stress induced by cyclopiazonic acid or dithiothreitol.^71^ The interaction between the LIR motifs and LC3 induces the formation of autophagosomes and their delivery to lysosomes.^74^ Thus, Sec62 plays an important, Sec61- and Sec63-independent, role during the compensation of ER stress—a process the authors described as recovER-phagy.^71^

Taken together, it was shown that Sec61 and Sec62 bear important functions beyond the protein translocation process, indicating that Sec proteins can influence intracellular signaling in various manners. How these additional functions are controlled in detail and how they are linked to other cellular signaling pathways remain elusive.

## Mutation and overexpression of *Sec* genes in human diseases

**Figure 3:**
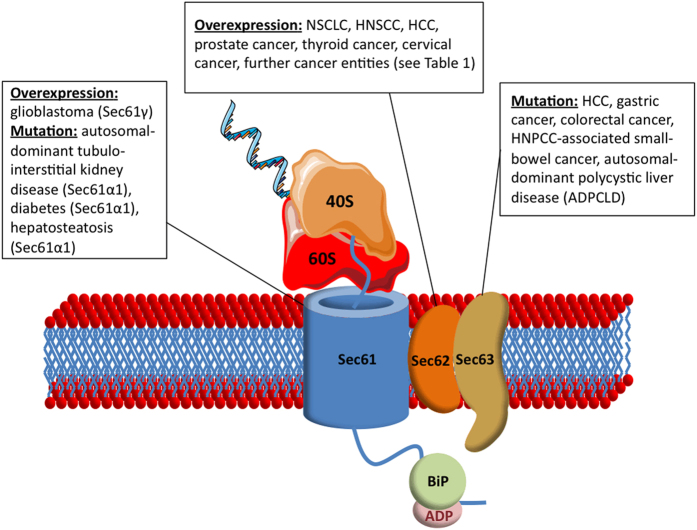
Overexpression and mutation of *SEC61*, *SEC62* and *SEC63* in human diseases. 40S, 40S ribosome subunit; 60S, 60S ribosome subunit; HCC, hepatocellular carcinoma; HNPCC, hereditary non-polyposis colorectal cancer; HNSCC, head and neck squamous cell carcinoma; NSCLC, non-small-cell lung cancer.

Over the past years, mutations, amplification and overexpression of *SEC61*, *SEC62* and *SEC63* have been linked to numerous human diseases (Figure 3). In 2004, Sec63 was the first human Sec protein being linked to a human disease, as Davila *et al.*^75^ showed that autosomal-dominant polycystic liver disease can be caused either by mutations in the *SEC63* gene or mutations in the protein kinase C substrate 80K-H gene (*PRKCSH*). For *SEC63*, two frameshift mutations, two nonsense mutations and two mutations predicted to disrupt splice donor–acceptor sites were described in a collective of 63 individuals, all leading to a loss of gene function. Further studies confirmed these results and strengthened the role of *SEC63* as a driver gene in the pathogenesis of autosomal-dominant polycystic liver disease by the disruption of co-translational transport of proteins, such as polycystins I and II, into the ER.^76–79^

Comparably, loss-of-function mutations have also been described for the *SEC61A1* gene and could be linked to autosomal-dominant tubulo-interstitial kidney disease in humans (ADTKD)^80^ as well as diabetes and hepatosteatosis in mice.^81^ Bolar *et al.* investigated renal tissue samples from two families with ADTKD and identified two different missense variants of *SEC61A1* (c.553A<G (p.Thr185Ala) and c.200T<G (p.Val67Gly)), whereas none of the otherwise frequently mutated genes *UMOD*, *MUC1* and *REN* were altered. The defective Sec61α1 variants were delocalized to the Golgi apparatus and, when induced in zebrafish embryos, led to convolution defects of the pronephric tubules consistent with the histological findings in ADTKD patients.

Another mutation of the *SEC61A1* gene (Y344H) was found to cause excessive ER stress and, as a consequence, inducing apoptosis of pancreatic ß-cells in C57BL/6 mice, which finally led to diabetes and hepatosteatosis. Thereby, transgenic ß-cell-specific expression of normal *SEC61A1* could rescue diabetes and ß-cell loss in mutant mice proving a critical role of Sec61α1 in the ß-cell response to glucose. One study introduced the mutant *SEC61A1* variant together with *SEC61A1* targeting siRNA into human cells and observed that the mutant Sec61 channel could not substitute for the wild-type channel with respect to BiP-dependent protein transport into the ER and cellular calcium homeostasis.^53^ Therefore, this study suggested that the mutated *SEC61A1* gene causes apoptosis of professional secretory cells such as ß-cells because of disturbed calcium homeostasis. However, no study has investigated so far the impact of this point mutation in human patients suffering from diabetes so that a direct link to human disease is still missing.

Apart from the mentioned kidney, liver and metabolic diseases, mutations and especially an amplification and overexpression of *SEC* genes were found to be frequent molecular characteristics of various human tumor diseases (Table 1). For *SEC63*, frameshift mutations caused by microsatellite instability were found in 37.5% of microsatellite-unstable gastric cancers,^82^ 48.8% of colorectal cancers,^82^ 56% of small-bowel cancer associated with hereditary non-polyposis colorectal cancer^83^ and in one case of hepatocellular carcinoma associated with Lynch syndrome.^84^ However, functional analyses to further uncover the role of Sec63 in human carcinogenesis were solely conducted by Casper *et al.*,^84^ who showed that a low hepatic expression of *SEC63* correlated with a decreased apoptosis rate and an increased proliferative activity of hepatocytes in BXD mice. Altogether, these studies indicate a potential role of *SEC63* as a tumor suppressor gene in the carcinogenesis of gastric cancer, colorectal cancer and hepatocellular cancer (HCC) without, however, providing a link of disrupted Sec63 function to tumor cell biology in these entities.

For *SEC61*, only one study reported a potential relevance in human cancer so far.^85^ In this study, copy-number changes and the messenger RNA (mRNA) expression of the Sec61γ-coding gene (*SEC61G*) were investigated in 43 human glioblastoma samples using quantitative PCR. Thereby, high copy-number gains (>4-fold) were found in 47% and an overexpression compared with healthy brain tissue in 77% of cases. Neither the genes coding for Sec61α (*SEC61A1*) nor for Sec61ß (*SEC61B*) showed comparably high expression levels with, however, a tendency to an elevated expression of both genes in glioblastoma samples compared with healthy brain tissue. When silencing the *SEC61G* gene in the human glioblastoma cell line H80, Lu *et al.* could observe reduced cell viability with an increased rate of apoptosis. Induction of ER stress by treating H80 and HeLa cells with tunicamycin—an inhibitor of N-linked glycosylation—led to an increase in *SEC61G* expression, indicating a potential role of Sec61γ in ER stress response. As several other studies reported a general ER expansion under conditions of ER stress,^86,87^ it is uncertain if Sec61γ has further functions during the compensation of ER stress apart from expanding ER capacity.

The strongest evidence for a causative role in cancer development and tumor cell biology exists for *SEC62* with the first association with human cancer having been reported in 2006: Jung *et al.*^88^ investigated copy-number changes in 22 prostate cancer samples and found copy-number gains of the *SEC62* gene in 50% of cases as well as increased *SEC62*-mRNA levels in all analyzed samples. Following the promising results of this study, Greiner *et al.*^89^ investigated *SEC62* expression at the protein level in 2071 tissue samples from 55 different tumor entities in an immunohistochemical multitissue tumor microarray. Thereby, 72% of all tumors showed detectable expression levels of *SEC62* with the highest percentage of an increased expression compared with healthy tissue from the same origin in lung cancer (93–97%, depending on the subtype) and thyroid cancer (87–100%, depending on the subtype). These results for lung and thyroid cancer were confirmed in a study by Linxweiler *et al.*,^90^ including 70 non-small-cell lung cancer cases and 10 thyroid cancer cases. Increased Sec62 protein and *SEC62*-mRNA levels compared with healthy lung tissue from the same patients were detected in 80% and 60.9% of cases, respectively. For thyroid cancer, increased Sec62 protein levels were observed in 40% and increased *SEC62*-mRNA levels in 60% of cases. A study focusing on *SEC62* expression in dysplastic cervical lesions^91^ found *SEC62* amplifications in 23% and increased Sec62 protein levels in 100% of cervical cancer cases with a gradually increasing *SEC62* expression depending on the severity of dysplasia. Wemmert *et al.*^92^ investigated the expression of *SEC62* in 35 cases of head and neck squamous cell carcinomas using immunohistochemistry and found a strong staining intensity in 34% and a moderate staining intensity in 23% of cases. Another study focused on the expression of *SEC62* in peripheral blood mononuclear cells from 80 HCC patients and 30 healthy individuals. Hereby, Sec62-mRNA and protein content were significantly higher in the blood samples from HCC patients compared with healthy controls.^93^

On the whole, all studies addressing the expression of *SEC62* in human cancer so far consistently reported an increased *SEC62* expression level for the majority of investigated cases both in the tumor tissue^88–92,94^ and in peripheral blood mononuclear cells,^93^ suggesting that *SEC62* plays a crucial role in the pathogenesis of various tumor entities and bears a potential oncogenic function. This hypothesis is further substantiated by the fact that the *SEC62*-encoding chromosomal region 3q26 is amplified in numerous human cancer entities including cervical cancer,^95,96^ non-small-cell lung cancer,^97^ esophageal cancer,^98^ ovarian cancer,^99^ and head and neck cancer.^100,101^ Hagerstrand *et al.*^102^ screened 3131 tumor samples from 26 different tumor entities for somatic copy-number alterations and found the *SEC62*-encoding region 3q26 to be amplified in 22% of cases. Indeed, a following systematic interrogation of 3q26 by gain- and loss-of-function studies identified *SEC62/TLOC1* as a ‘tumor-driver gene’ encoded in this region. The same group investigated the effect of short hairpin RNA-mediated *SEC62* knockout on the proliferation of 16 different human cell lines and found that cell lines harboring an 3q26 amplification rely on *SEC62* for normal proliferative activity. Moreover, *SEC62* overexpression increased anchorage-independent growth in human mammary epithelial cells and induced subcutaneous tumor growth of otherwise non-tumor-forming murine embryo fibroblasts (NIH3T3) in C.Cg/AnNTac-Foxn1^nunu^ mice^102^ again pointing to a potential oncogenic function of *SEC62*.

To identify further roles of Sec62 in tumor cell biology apart from the known function in protein translocation across the ER membrane, several studies investigated the effect of *SEC62* silencing and *SEC62* overexpression on neoplastic and non-neoplastic human cell lines. Consistently, a *SEC62* knockdown markedly reduced the migration and invasion potential of prostate cancer cells^90^ as well as the migration of NSCLC cells,^90^ thyroid cancer cells^90^ and cervical cancer cells,^91^ whereas *SEC62* overexpression stimulated the migration of cervical cancer cells^91^ and even human embryonic kidney cells.^90^ The latter is particularly telling since it provided a direct link between Sec62 and cell migration. Of note, neither *SEC62* silencing nor *SEC62* overexpression markedly affected cell proliferation in these studies.^89–91^ However, other studies reported an impairment of cell proliferation in Sec62-depleted cell lines harboring a 3q26 amplification^102^ as well as in Sec62-depleted HeLa cells.^32^ Though all of these studies indicate a crucial role of *SEC62* in cancer cell migration and invasion—molecular processes that are essential for tumor metastasis—it is not yet clear how this function of Sec62 is mediated on the molecular level. As Sec62 is involved in the protein translocation process at the ER, it is conceivable that a distinct subset of migration-relevant precursor proteins rely on Sec62 for efficient transport. However, no according substrates have been identified so far. Linxweiler *et al.*^91^ investigated a potential role of *SEC62* in the induction of epithelial–mesenchymal transition, a highly conserved molecular process that is essential for metastasis formation, but found no influence of Sec62 on the expression of epithelial–mesenchymal transition markers as vimentin and E-cadherin. Apart from that, the inhibitory effect of high *SEC62* expression levels on the Sec61-mediated Ca^2+^ efflux from the ER lumen^68^ represents a possible connection between Sec62 and cell migration. Though the exact molecular mechanisms linking Sec62 and the Ca^2+^ efflux as well as Ca^2+^ and cellular migration remain elusive.

In addition to an increased potential for migration and invasion, *SEC62* overexpressing cancer cells were found to exhibit a higher tolerance to cellular stress, such as thapsigargin (TG)-induced ER stress,^89,90^ another hallmark of cancer cells. Again, increased calcium stress tolerance could be induced by *SEC62* overexpression in human embryonic kidney cells^90^ and was reduced by *SEC62* knockdown from cancer cells, thereby providing a direct link between *SEC62* overexpression and tolerance to cellular calcium stress (see below).

Taken together, functional analyses investigating the role of Sec62 in tumor cell biology have shown that tumor cells could profit from an increased *SEC62* expression level in terms of an increased capability to migrate and invade the surrounding tissue, which is essential for the formation of metastases. In addition, the recently observed function of Sec62 in the recovery from ER stress^71^ represents a further potentially beneficial effect of high *SEC62* expression levels for tumor cells and maybe linked to the role of Sec62 in stress tolerance.

## Sec proteins as molecular biomarkers and therapeutic targets in human cancer

To explore the clinical relevance of Sec62 as a potential biomarker especially in tumor diseases, the *SEC62* expression level of the respective tumor tissue as well as peripheral blood mononuclear cells was correlated with the patients’ clinical data, including TNM stage and survival in several of the before-mentioned studies. Hereby, Greiner *et al.*^94^ found a correlation between the Sec62 protein levels in prostate cancer tissue with the patients’ Gleason score—a histopathological grading system with high scores indicating a poor prognosis—indicating a worse outcome for patients with higher Sec62 levels. Comparably, the Sec62 protein levels in NSCLC as well as head and neck squamous cell carcinomas tissue samples significantly correlated with a shorter overall survival.^68,92^ For NSCLC patients, an additional correlation of high Sec62 levels with a dedifferentiation of the tumors and the occurrence of lymph node metastases was found,^90^ again pointing to a potential function of Sec62 in tumor metastasis. In HCC patients, a high expression of *SEC62* in peripheral blood mononuclear cells correlated with a reduced recurrence-free survival characterizing Sec62 as an independent predictor of HCC recurrence.^93^ In contrast, prostate cancer patients with *SEC62* copy-number gains showed a lower risk of and a longer time to progression compared with patients without *SEC62* copy-number gains in the study by Jung *et al.*^88^ Considering the comparably low number of patients in this study (*n*=22) and the consistent findings of the other studies, Sec62 seems to be an independent biomarker for the patients’ prognosis in several human cancers. To confirm this potential role of Sec62 as a prognostic marker and to establish a valid basis for clinical applications, further tumor entities enclosing larger patient cohorts have to be investigated in future studies.

Though Sec61 and Sec63 have been linked to human diseases as well, a potential function of these proteins as diagnostic or prognostic biomarkers seems unlikely, as all diseases showing *SEC61A1* and *SEC63* mutations can also be caused by other genetic alterations.^80,81,103^ In the context of human cancer, the percentage of gastric cancer,^82^ small-bowel cancer^83^ and colorectal cancer cases^82^ showing frameshift mutations of *SEC63* as well as the percentage of glioblastoma cases showing a *SEC61G* amplification and overexpression^85^ is too low to allow diagnostic conclusions. A potential correlation of *SEC63* and *SEC61G* expression levels with the patients’ survival has not been investigated so far.

**Figure 4:**
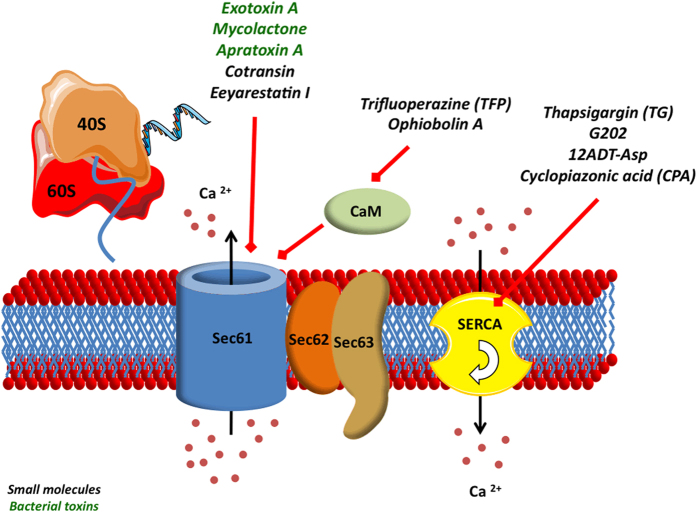
Protein translocation complex as a target of bacterial toxins and small molecule therapeutics. Small molecules are written in italic and black letters; bacterial toxins in italic and green letters. The red arrows indicate inhibitory effects directed against the respective target structure. 40S, 40S ribosome subunit; 60S, 60S ribosome subunit; CaM, calmodulin; SERCA, sarcoplasmic/endoplasmic reticulum Ca^2+^-ATPase.

Regarding the relevance of Sec62 as a prognostic biomarker in different cancer entities and the stimulation of tumor cell migration and invasion by high *SEC62* expression levels in cell culture experiments, one can hypothesize that a suppression of *SEC62* gene function could possibly influence tumor metastasis *in vivo* too. Unfortunately, there are many known synthetic (eeyarestatin I and cotransin) and natural inhibitors (exotoxin A, mycolactone and apratoxin A) of the Sec61 channel (Figure 4),^104–109^ but no direct inhibitors of Sec62 and Sec63. However, it is possible to antagonize the function of Sec62 in cellular Ca^2+^ homeostasis by either inhibiting the sarcoplasmic/ER Ca^2+^ ATPase and thereby decreasing the active Ca^2+^ import from the cytosol into the ER lumen, or antagonizing the CaM-mediated Sec61 closure with CaM antagonists and thereby increasing the passive Ca^2+^ efflux from the ER lumen through the Sec61 channel (Figure 4). Linxweiler *et al.*^68^ performed *in vitro* experiments to investigate a possible phenocopy of *SEC62* gene silencing in human tumor cells by the treatment with CaM antagonists. Indeed, the CaM antagonists trifluoperazine (TFP) and ophiobolin A induced a dose-dependent inhibition of tumor cell migration, an additional inhibition of tumor cell proliferation at higher concentrations and sensitized the cells to TG)-induced ER stress—the same effects that were reported for Sec62-depleted tumor cells.^89–91^ Hence, a treatment with CaM antagonists represents a potential mechanism how to achieve a functional *SEC62* knockdown in a living organism and thereby inhibiting the migratory and proliferative potential of tumor cells. As TFP was used for the treatment of patients with psychiatric disorders over many years^110^ and one retrospective study observed a beneficial side effect of TFP treatment on the clinical course of tumor diseases,^111^ TFP appears to be a promising new substance for a targeted therapy approach, especially in tumors with high *SEC62* expression levels. In addition, the observation that low Sec62 levels sensitize tumor cells to TG-induced ER stress^68,89^ is of potential clinical relevance. Over the past years, several *in vitro* and *in vivo* studies investigated the applicability of TG and TG prodrugs (G202 and 12ADT-Asp) for the treatment of cancer patients with promising results.^112–114^ In addition, a first clinical phase I trial including 44 patients with locally advanced or metastatic solid tumors was finished in august 2013 (‘dose-escalation phase I study of G202 in patients with advanced solid tumors’, clinical trials gov. identifier NCT01056029) and reported an acceptable tolerability and favorable pharmacokinetic profile for G202, also called mipsagargin.^115^ For this treatment approach, Sec62 could serve as a predictive biomarker, as it was shown that high *SEC62* expression levels attenuate the therapeutic efficacy of sarcoplasmic/ER Ca^2+^ ATPase inhibitors.^68,89^ With regard to the molecular background, a combined treatment of cancer cells with a sarcoplasmic/ER Ca^2+^ ATPase inhibitor and a CaM antagonist could overcome Sec62-mediated resistance and show synergistic therapeutic effects^68^—a new therapy approach that has to be evaluated in future studies.

Taken together, studies of the past decades have not only further illuminated the detailed mechanism of protein translocation into and across the ER membrane but gave also first insights into the role of proteins Sec61, Sec62 and Sec63 in human diseases including cancer, diabetes, liver and kidney diseases. Further investigations of the pathophysiological relevance of Sec proteins have the potential to provide a better understanding of disease emergence and progression, to enable a better prognostication and therapy planning in various cancer entities and to uncover new therapeutic targets.

## Figures and Tables

**Table 1:** Sec proteins in human cancer

*Protein*	*Study*	*Tumor entity*	*No. of patients*	*Animal model*	*Cell lines*	*Findings*
Sec61γ	Lu *et al.*^85^	Glioblastoma	*N*=59	/	H80, HeLa	*SEC61G* is amplified and overexpressed in glioblastomas; *SEC61G* silencing suppresses cell growth and induces apoptosis; ER stress induces *SEC61G* expression
Sec62	Jung *et al.*^88^	Prostate cancer	*N*=22	/	PC3, U145, DU145MN1	*SEC62* copy-number gains in 50% of all prostate cancer samples+increased Sec62 protein level; however, copy-number gain in patients with lower risk of and longer time to progression
	Greiner *et al.*^89^	55 different tumor entities	*N*=2071	/	DU145, PC3, LNCaP	*SEC62* silencing sensitizes DU145, PC3 and LNCaP cells to thapsigargin treatment; correlation of thapsigargin sensitivity with *SEC62* expression in DU145, PC3 and LNCaP cells; 72% of all tumors show *SEC62* expression; *SEC62* overexpression in tumor tissue compared with healthy tissue of the same organ in lung cancer (93–97%) and thyroid cancer (87–100%); *SEC62* silencing reduces the migration of all tested cell lines
	Greiner *et al.*^94^	Prostate cancer	*N*=32	/	A549, H1299, HT1080, TX3868, PC3	*SEC62* overexpression in the majority of prostate cancer cases correlating with the Gleason score
	Linxweiler *et al.*^90^	NSCLC thyroid cancer	*N*=70 *N*=10	/ /	A549, BC01, BHT101, ML1, HEK293	*SEC62* overexpression in tumor tissue compared with healthy lung tissue; high expression levels correlate lymph node metastases and poor tumor differentiation; *SEC62* silencing inhibits the migration of NSCLC cells; increased Sec62 protein (40%) and mRNA (60%) levels in thyroid cancer compared with tumor-free tissue; *SEC62* silencing inhibits the migration of BC01, BHT101 and ML1 cells, and sensitizes the cells to thapsigargin-induced ER stress; *SEC62* overexpression stimulates the migration of HEK293 cells
	Weng *et al.*^93^	HCC	*N*=110	/	/	High *SEC62* expression in PBMCs correlates with reduced recurrent-free survival; Sec62 as an independent predictor of HCC recurrence
	Linxweiler *et al.*^68^	NSCLC	*N*=70	/	PC3, HeLa, A549, BC01, BHT101, ML1, HEK293	High *SEC62* expression correlates with a poorer OS; effect of *SEC62* silencing on tumor cell migration and ER stress tolerance can be mimicked by CaM anatgonists; *SEC62* overexpression in HEK293 cells increases ER stress tolerance
	Hagerstrand *et al.*^102^	26 different tumor entities	*N*=3131	C.Cg/AnNTac-Foxn1^nunu^ mice	T47D, HCC1937, H3255, HCC95, H1819, H26, TE6, RPMI8226, Fu-Ov-O1, COLO320, MCF7, MDA-MB-231, ZR75-1, HMEC, HCC364, DLD1, HMLE	3q26 amplification: 22% of tumor samples (43.7% in ovarian cancer, 31.7% in breast cancer, 31.2% in non-small-cell lung cancer). 3q26-encoded *SEC62* is required for the proliferation of celll lines with 3q26 amplification; *SEC62* overexpression in HMLE cells induces subcutaneous tumor growth in C.Cg/AnNTac-Foxn1^nunu^ mice; *SEC62* expression level correlates wtih 3q26 amplification; *SEC62* as a tumor-driver gene of the 3q26 region
	Wemmert *et al.*^92^	HNSCC	*N*=35	/	/	High Sec62 protein level is associated with poorer OS and PFS
	Linxweiler *et al.*^91^	Cervical cancer	*N*=107	/	HeLa, MCF7	Stepwise increase in *SEC62* expression depending on the severity of dysplasia with the highest expression in invasive cervical cancer; *SEC62* silencing inhibits and *SEC62* overexpression stimulates the migration of cervical cancer cells
Sec63	Mori *et al.*^82^	Gastric cancer CRC	*N*=16 *N*=43	/	/	Frameshift mutations of the *SEC63* gene due to microsatellite instability in 37.5% of gastric cancers and 48.8% of colorectal cancers
	Schulmann *et al.*^83^	HNPCC-associated SBC	*N*=17	/	/	Frameshift mutations of the *SEC63* gene due to microsatellite instaibility in 56% of HNPCC-associated small-bowel cancers
	Casper *et al.*^84^	HCC	*N*=1	BXD mice	/	Microsatellite instability in the *SEC63* gene in the tested HCC case; correlation of low hepatic *SEC63* expression with decreased apoptosis and increased proliferation rate in the mouse model

Abbreviations: CRC, colorectal cancer; HCC, hepatocellular cancer; HMLE, human mammary epithelial; HNSCC, head and neck squamous cell carcinoma; HNPCC, hereditary non-polyposis colorectal cancer; NSCLC, non-small-cell lung cancer; OS, overall survival, PBMC, peripheral blood mononuclear cell; PFS, progression-free survival, SBC, small-bowel cancer; *SEC61G*, Sec61γ-coding gene.

## References

[bib1] Palade G. Intracellular aspects of the process of protein synthesis. Science 1975; 189: 347–358. 1096303 10.1126/science.1096303

[bib2] Zimmermann R, Eyrisch S, Ahmad M, Helms V. Protein translocation across the ER membrane. Biochim Biophys Acta 2011; 1808: 912–924. 20599535 10.1016/j.bbamem.2010.06.015

[bib3] Borgese N, Fasana E. Targeting pathways of C-tail-anchored proteins. Biochim Biophys Acta 2011; 1808: 937–946. 20646998 10.1016/j.bbamem.2010.07.010

[bib4] Rapoport TA, Rolls MM, Jungnickel B. Approaching the mechanism of protein transport across the ER membrane. Curr Opin Cell Biol 1996; 8: 499–504. 8791447 10.1016/s0955-0674(96)80027-5

[bib5] Matlack KE, Mothes W, Rapoport TA. Protein translocation: tunnel vision. Cell 1998; 92: 381–390. 9476897 10.1016/s0092-8674(00)80930-7

[bib6] Schlenstedt G, Zimmermann R. Import of frog prepropeptide GLa into microsomes requires ATP but does not involve docking protein or ribosomes. EMBO J 1987; 6: 699–703. 3034606 10.1002/j.1460-2075.1987.tb04810.x PMC553453

[bib7] Kutay U, Ahnert-Hilger G, Hartmann E, Wiedenmann B, Rapoport TA. Transport route for synaptobrevin via a novel pathway of insertion into the endoplasmic reticulum membrane. EMBO J 1995; 14: 217–223. 7835332 10.1002/j.1460-2075.1995.tb06994.x PMC398073

[bib8] Walter P, Blobel G. Purification of a membrane-associated protein complex required for protein translocation across the endoplasmic reticulum. Proc Natl Acad Sci USA 1980; 77: 7112–7116. 6938958 10.1073/pnas.77.12.7112 PMC350451

[bib9] Walter P, Ibrahimi I, Blobel G. Translocation of proteins across the endoplasmic reticulum. I. Signal recognition protein (SRP) binds to *in-vitro*-assembled polysomes synthesizing secretory protein. J Cell Biol 1981; 91: 545–550. 7309795 10.1083/jcb.91.2.545 PMC2111968

[bib10] Görlich D, Rapoport TA. Protein translocation into proteoliposomes reconstituted from purified components of the endoplasmic reticulum membrane. Cell 1993; 75: 615–630. 8242738 10.1016/0092-8674(93)90483-7

[bib11] Römisch K, Webb J, Herz J, Prehn S, Frank R, Vingron M et al. Homology of 54 K protein of signal-recognition particle, docking protein and two E. coli proteins with putative GTP-binding domains. Nature 1989; 340: 478–482. 2502717 10.1038/340478a0

[bib12] Rapiejko PJ, Gilmore R. Empty site forms of the SRP54 and SR alpha GTPases mediate targeting of ribosome-nascent chain complexes to the endoplasmic reticulum. Cell 1997; 89: 703–713. 9182758 10.1016/s0092-8674(00)80253-6

[bib13] Mitra K, Frank J. A model for co-translational translocation: ribosome-regulated nascent polypeptide translocation at the protein-conducting channel. FEBS Lett 2006; 580: 3353–3360. 16714018 10.1016/j.febslet.2006.05.019

[bib14] Tyedmers J, Lerner M, Wiedmann M, Volkmer J, Zimmermann R. Polypeptide-binding proteins mediate completion of co-translational protein translocation into the mammalian endoplasmic reticulum. EMBO Rep 2003; 4: 505–510. 12704426 10.1038/sj.embor.embor826 PMC1319181

[bib15] Alder NN, Shen Y, Brodsky JL, Hendershot LM, Johnson AE. The molecular mechanisms underlying BiP-mediated gating of the Sec61 translocon of the endoplasmic reticulum. J Cell Biol 2005; 168: 389–399. 15684029 10.1083/jcb.200409174 PMC2171714

[bib16] Dudek J, Benedix J, Cappel S, Greiner M, Jalal C, Müller L et al. Functions and pathologies of BiP and its interaction partners. Cell Mol Life Sci 2009; 66: 1556–1569. 19151922 10.1007/s00018-009-8745-y PMC11131517

[bib17] Nguyen TH, Law DT, Williams DB. Binding protein BiP is required for translocation of secretory proteins into the endoplasmic reticulum in Saccharomyces cerevisiae. Proc Natl Acad Sci USA 1991; 88: 1565–1569. 1996357 10.1073/pnas.88.4.1565 PMC51060

[bib18] Sanders SL, Schekman R. Polypeptide translocation across the endoplasmic reticulum membrane. J Biol Chem 1992; 267: 13791–13794. 1321124

[bib19] Brodsky JL, Schekman R. A Sec63p-BiP complex from yeast is required for protein translocation in a reconstituted proteoliposome. J Cell Biol 1993; 123: 1355–1363. 8253836 10.1083/jcb.123.6.1355 PMC2290880

[bib20] Panzner S, Dreier L, Hartmann E, Kostka S, Rapoport TA. Posttranslational protein transport in yeast reconstituted with a purified complex of Sec proteins and Kar2p. Cell 1995; 81: 561–570. 7758110 10.1016/0092-8674(95)90077-2

[bib21] Lyman SK, Schekman R. Interaction between BiP and Sec63p is required for the completion of protein translocation into the ER of Saccharomyces cerevisiae. J Cell Biol 1995; 131: 1163–1171. 8522580 10.1083/jcb.131.5.1163 PMC2120636

[bib22] Matlack KES, Plath K, Misselwitz B, Rapoport TA. Protein transport by purified yeast Sec complex and Kar2p without membranes. Science 1997; 277: 938–941. 9252322 10.1126/science.277.5328.938

[bib23] Lang S, Benedix J, Fedeles SV, Schorr S, Schirra C, Schäuble N et al. Different effects of Sec61α, Sec62 and Sec63 depletion on transport of polypeptides into the endoplasmic reticulum of mammalian cells. J Cell Sci 2012; 125: 1958–1969. 22375059 10.1242/jcs.096727 PMC4074215

[bib24] Weitzmann A, Baldes C, Dudek J, Zimmermann R. The heat shock protein 70 molecular chaperone network in the pancreatic endoplasmic reticulum—a quantitative approach. FEBS J 2007; 274: 5175–5187. 17850331 10.1111/j.1742-4658.2007.06039.x

[bib25] Blobel G, Dobberstein B. Transfer of proteins across membranes. I. Presence of proteolytically processed and unprocessed nascent immunoglobulin light chains on membrane-bound ribosomes of murine myeloma. J Cell Biol 1975; 67: 835–851. 811671 10.1083/jcb.67.3.835 PMC2111658

[bib26] Knauer R, Lehle L. The oligosaccharyltransferase complex from yeast. Biochim Biophys Acta 1999; 1426: 259–273. 9878773 10.1016/s0304-4165(98)00128-7

[bib27] Ng DT, Brown JD, Walter P. Signal sequences specify the targeting route to the endoplasmic reticulum membrane. J Cell Biol 1996; 134: 169–178. 10.1083/jcb.134.2.269 PMC2120870 8707814

[bib28] Schlenstedt G, Gudmundsson GH, Boman HG, Zimmermann R. A large presecretory protein translocates both cotranslationally, using signal recognition particle and ribosome, and post-translationally, without these ribonucleoparticles, when synthesized in the presence of mammalian microsomes. J Biol Chem 1990; 265: 13960–13968. 2380197

[bib29] Sagstetter M, Zimmermann R. Assembly of M13 and M13am8H1R1 procoat protein into microsomes is stimulated by rabbit reticulocyte lysate and ATP. Biochem Biophys Res Commun 1988; 153: 498–501. 3289534 10.1016/s0006-291x(88)81122-7

[bib30] Wiech H, Stuart R, Zimmermann R. Role of cytosolic factors in the transport of proteins across membranes. Semin Cell Biol 1990; 1: 55–63. 1983271

[bib31] Brodsky JL, Goeckeler J, Schekman R. BiP and Sec63p are required for both co- and posttranslational protein translocation into the yeast endoplasmic reticulum. Proc Natl Acad Sci USA 1995; 92: 9643–9646. 7568189 10.1073/pnas.92.21.9643 PMC40858

[bib32] Abell BM, Pool MR, Schlenker O, Sinning I, High S. Signal recognition particle mediates post-translational targeting in eukaryotes. EMBO J 2004; 23: 2755–2764. 15229647 10.1038/sj.emboj.7600281 PMC514945

[bib33] Lakkaraju AK, Thankappan R, Mary C, Garrison JL, Taunton J, Strub K. Efficient secretion of small proteins in mammalian cells relies on Sec62-dependent posttranslational translocation. Mol Biol Cell 2012; 23: 2712–2722. 22648169 10.1091/mbc.E12-03-0228 PMC3395660

[bib34] Conti BJ, Devaraneni PK, Yang ZY, David LL, Skach WR. Cotranslational stabilization of Sec62/63 in the ER Sec61 translocon is controlled by distinct substrate-driven translocation events. Mol Cell 2015; 58: 269–283. 25801167 10.1016/j.molcel.2015.02.018 PMC4402133

[bib35] Tyedmers J, Lerner M, Bies C, Dudek J, Skowronek MH, Haas IG et al. Homologues of the yeast Sec complex subunits Sec62p and Sec63p are abundant proteins in dog pancreas microsomes. Proc Natl Acad Sci USA 2000; 97: 7214–7219. 10860986 10.1073/pnas.97.13.7214 PMC16525

[bib36] Görlich D, Prehn S, Hartmann E, Kalies KU, Rapoport TA. A mammalian homolog of SEC61p and SECYp is associated with ribosomes and nascent polypeptides during translocation. Cell 1992; 71: 489–503. 1423609 10.1016/0092-8674(92)90517-g

[bib37] Wirth A, Jung M, Bies C, Frien M, Tyedmers J, Zimmermann R et al. The Sec61p complex is a dynamic precursor activated channel. Mol Cell 2003; 12: 261–268. 12887911 10.1016/s1097-2765(03)00283-1

[bib38] Van den Berg B, Clemons Jr WM, Collinson I, Modis Y, Hartmann E, Harrison SC et al. X-ray structure of a protein-conducting channel. Nature 2004; 427: 36–44. 14661030 10.1038/nature02218

[bib39] Becker T, Bushan S, Jarasch A, Armache JP, Funes S, Jossinet F et al. Structure of monomeric yeast and mammalian Sec61 complexes interacting with the translating ribosome. Science 2009; 326: 1369–1373. 19933108 10.1126/science.1178535 PMC2920595

[bib40] Pfeffer S, Dudek J, Gogala M, Schorr S, Linxweiler J, Lang S et al. Structure of the mammalian oligosaccharyl-transferase complex in the native ER protein translocon. Nat Commun 2014; 5: 3072. 24407213 10.1038/ncomms4072

[bib41] Pfeffer S, Burbaum L, Unverdorben P, Pech M, Chen Y, Zimmermann R et al. Structure of native Sec61 protein-conducting channel. Nat Commun 2015; 6: 8403. 26411746 10.1038/ncomms9403 PMC4598622

[bib42] Meyer HA, Grau H, Kraft R, Kostka S, Prehn S, Kalies KU et al. Mammalian Sec61 is associated with Sec62 and Sec63. J Biol Chem 2000; 275: 14550–14557. 10799540 10.1074/jbc.275.19.14550

[bib43] Müller L, de Escauriaza MD, Lajoie P, Theis M, Jung M, Müller A et al. Evolutionary gain of function for the ER membrane protein Sec62 from yeast to humans. Mol Biol Cell 2010; 21: 891–903. 10.1091/mbc.E09-08-0730 PMC2828957 20071467

[bib44] Rapoport TA. Protein translocation across the eukaryotic endoplasmic reticulum and bacterial plasma membranes. Nature 2007; 450: 663–669. 18046402 10.1038/nature06384

[bib45] Rapoport TA. Protein transport across the endoplasmic reticulum membrane. FEBS J 2008; 275: 4471–4478. 18671729 10.1111/j.1742-4658.2008.06588.x

[bib46] Hartmann E, Sommer T, Prehn S, Gorlich D, Jentsch S, Rapoport TA. Evolutionary conservation of commponents of the protein translocation complex. Nature 1994; 367: 654–657. 8107851 10.1038/367654a0

[bib47] Deshaies RJ, Schekman R. SEC62 encodes a putative membrane protein required for protein translocation into the yeast endoplasmic reticulum. J Cell Biol 1989; 109: 2653–2664. 2687286 10.1083/jcb.109.6.2653 PMC2115948

[bib48] Deshaies RJ, Schekman R. Structural and functional dissection of Sec62p, a membrane-bound somponent of the yeast endoplasmic reticulum protein import machinery. Mol Cell Biol 1990; 10: 6024–6035. 2233730 10.1128/mcb.10.11.6024-6035.1990 PMC361400

[bib49] Deshaies RJ, Sanders SL, Feldheim DA, Schekman R. Assembly of yeast Sec proteins involved in translocation into the endoplasmic reticulum into a membrane-bound multisubunit complex. Nature 1991; 349: 806–808. 2000150 10.1038/349806a0

[bib50] Daimon M, Susa S, Suzuki K, Kato T, Yamatani K, Sasaki H. Identification of a human cDNA homologue to the Drosophila translocation protein 1 (Dtrp1). Biochem Biophys Res Commun 1997; 230: 100–104. 9020021 10.1006/bbrc.1996.5892

[bib51] Lyman SK, Schekman R. Binding of secretory precursor polypeptides to a translocon subcomplex is regulated by BiP. Cell 1997; 88: 85–96. 9019409 10.1016/s0092-8674(00)81861-9

[bib52] Plath K, Mothes W, Wilkinson BM, Stirling CJ, Rapoport TA. Signal sequence recognition in posttranslational protein transport across the yeast ER membrane. Cell 1998; 94: 795–807. 9753326 10.1016/s0092-8674(00)81738-9

[bib53] Schäuble N, Lang S, Jung M, Cappel S, Schorr S, Ulucan Ö et al. BiP-mediated closing of the Sec61 channel limits Ca2+ leakage from the ER. EMBO J 2012; 31: 3282–3296. 22796945 10.1038/emboj.2012.189 PMC3411083

[bib54] Simon SM, Blobel G. A protein-conducting channel in the endoplasmic reticulum. Cell 1991; 65: 371–380. 1902142 10.1016/0092-8674(91)90455-8

[bib55] Lomax RB, Camello C, Van Coppenolle F, Petersen OH. Basal and physiological Ca2+ leak from the endoplasmic reticulum of pancreatic acinar cells. Second messenger-activated channels and translocons. J Biol Chem 2002; 277: 26479–26485. 11994289 10.1074/jbc.M201845200

[bib56] Roy A, Wonderlin WF. The permeability of the endoplasmic reticulum is dynamically coupled to protein synthesis. J Biol Chem 2003; 278: 4397–4403. 12458217 10.1074/jbc.M207295200

[bib57] Van Coppenolle F, Vanden Abeele F, Slomianny C, Flourakis M, Hesketh J, Dewailly E et al. Ribosome-translocon complex mediates calcium leakage fom endoplasmic reticulum stores. J Cell Sci 2004; 117: 4135–4142. 15280427 10.1242/jcs.01274

[bib58] Flourakis M, Van Coppenolle F, Lehen’kyi V, Beck B, Skryma R. Passive calcium leak via translocon is a first step for iPLA2-pathway regulated store operated channels activation. FASEB J 2006; 20: 1215–1217. 16611832 10.1096/fj.05-5254fje

[bib59] Giunti R, Gamberucci A, Fulceri R, Banhegyi G. Both translocon and a cation channel are involved in the passive Ca2+ leak from the endoplasmic reticulum: a mechanistic study on rat liver microsomes. Arch Biochem Biophys 2007; 462: 115–121. 17481572 10.1016/j.abb.2007.03.039

[bib60] Ong HL, Liu X, Sharma A, Hedge RS, Ambudkar IS. Intracellular Ca2+ release via the ER translocon activates store-operated calcium entry. Pflugers Arch 2007; 453: 797–808. 17171366 10.1007/s00424-006-0163-5

[bib61] Wonderlin WF. Constitutive, translation-independent opening of the protein-conducting channel in the endoplasmic reticulum. Pflugers Arch 2009; 457: 917–930. 18604553 10.1007/s00424-008-0545-y

[bib62] Wuytack F, Racymackers L, Missiaen L. Molecular physiology of the SERCA and SPCA pumps. Cell Calcium 2002; 32: 279–305. 12543090 10.1016/s0143416002001847

[bib63] Lang S, Erdmann F, Jung M, Wagner R, Cavaliè A, Zimmermann R. Sec61 complexes form ubiquitous ER Ca2+ leak channels. Channels 2011; 5: 228–235. 21406962 10.4161/chan.5.3.15314

[bib64] Erdmann F, Schäuble N, Lang S, Jung M, Honigmann A, Ahmad M et al. Interaction of calmodulin with Sec61α limits Ca2+ leakage from the endoplasmic reticulum. EMBO J 2011; 30: 17–31. 21102557 10.1038/emboj.2010.284 PMC3020109

[bib65] Huang JB, Kindzelskii AL, Clark AJ, Petty HR. Identification of channels promoting calcium spikes and waves in HT1080 tumor cells: their apparent roles in cell motility and invasion. Cancer Res 2004; 64: 2482–2489. 15059902 10.1158/0008-5472.can-03-3501

[bib66] Scorrano L, Oakes SA, Opferman JT, Cheng EH, Sorcinelli MD, Pozzan T et al. BAX and BAK regulation of endoplasmic reticulum Ca2+: a control point for apoptosis. Science 2003; 300: 135–139. 12624178 10.1126/science.1081208

[bib67] Berridge MJ. The endoplasmic reticulum: a multifunctional signaling organelle. Cell Calcium 2002; 32: 235–249. 12543086 10.1016/s0143416002001823

[bib68] Linxweiler M, Schorr S, Schäuble N, Jung M, Linxweiler J, Langer F et al. Targeting cell migration and the endoplasmic reticulum stress response with calmodulin antagonists: a clinically tested small molecule phenocopy of SEC62 gene silencing in human tumor cells. BMC Cancer 2013; 13: 574. 24304694 10.1186/1471-2407-13-574 PMC3878975

[bib69] Crowley KS, Liao S, Worrell VE, Reinhart GD, Johnson AE. Secretory proteins move through the endoplasmic reticulum membrane via an aqueous, gated pore. Cell 1994; 78: 461–471. 8062388 10.1016/0092-8674(94)90424-3

[bib70] Walter P, Ron D. The unfolded protein response: from stress pathway to homeostatic regulation. Science 2011; 33: 1081–1086. 10.1126/science.1209038 22116877

[bib71] Fumagalli F, Noack J, Bergmann TJ, Cebollero E, Pisoni GB, Fasana E et al. Translocon component Sec62 acts in endoplasmic reticulum turnover during stress recovery. Nat Cell Biol 18: 1173–1184. 10.1038/ncb3423 27749824

[bib72] Guerriero CJ, Brodsky JL. The delicate balance between secreted protein folding and endoplasmic reticulum-associated degradation in human physiology. Physiol Rev 2012; 92: 537–576. 22535891 10.1152/physrev.00027.2011 PMC4162396

[bib73] Pisoni G, Molinari M. Five questions (with their answers) on ER-associated degradation. Traffic 2016; 17: 341–350. 27004930 10.1111/tra.12373

[bib74] Birgisdottir AB, Lamark T, Johansen T. The LIR motif—crucial for selective autophagy. J Cell Sci 2013; 126: 3237–3247. 23908376 10.1242/jcs.126128

[bib75] Davila S, Furu L, Gharavi AG, Tin X, Onoe T, Qian Q et al. Mutations in SEC63 cause autosomal dominant polycystic liver disease. Nat Genet 2004; 36: 575–577. 15133510 10.1038/ng1357

[bib76] Drenth JP, Martina JA, van de Kerkhof R, Bonifacino JS, Jansen JB. Polycystic liver disease is a disorder of cotranslational protein processing. Trends Mol Med 2005; 11: 37–42. 15649821 10.1016/j.molmed.2004.11.004

[bib77] Waanders E, te Morsche RHM, de Man RA, Jansen JBMJ, Drenth JPH. Extensive mutational analysis of *PRKCSH* and *SEC63* broadens the spectrum of polycystic liver disease. Hum Mutat 2006; 27: 830. 10.1002/humu.9441 16835903

[bib78] Janssen MJ, Salomon J, te Morsche RHM, Drenth JPH. Loss of heterozygosity is present in *SEC63* germline carriers eith polycystic liver disease. PLoS One 2012; 7: e50324. 23209713 10.1371/journal.pone.0050324 PMC3508994

[bib79] Fedeles SV, Gallagher AR, Somlo S. Polycystin-1: a master regulator of intersecting cystic pathways. Trends Mol Med 2014; 20: 251–260. 24491980 10.1016/j.molmed.2014.01.004 PMC4008641

[bib80] Bolar NA, Golzio C, Živná M, Hayot G, Van Hemelrijk C, Schepers D et al. Heterozygous loss-of-function *SEC61A1* mutations cause autosomal-dominant tubulo-interstitial and glomerulocystic kidney disease with anemia. Am J Hum Genet 2016; 99: 174–187. 27392076 10.1016/j.ajhg.2016.05.028 PMC5005467

[bib81] Lloyd DJ, Wheeler MC, Gekakis N. A point mutation in Sec61α1 leads to diabetes and hepatosteatosis in mice. Diabetes 2010; 59: 460–470. 19934005 10.2337/db08-1362 PMC2809972

[bib82] Mori Y, Sato F, Selaru FM, Olaru A, Perry K, Kimos MC et al. Instabilotyping reveals unique mutational spectra in microsatellite-unstable gastric cancer. Cancer Res 2002; 62: 3641–3645. 12097267

[bib83] Schulmann K, Brasch FE, Kunstmann E, Engel C, Pagenstecher C, Vogelsang H et al. HNPCC-associated small bowel cancer: clinical and molecular characteristics. Gastroenterology 2005; 128: 590–599. 15765394 10.1053/j.gastro.2004.12.051

[bib84] Casper M, Weber SN, Kloor M, Müllenbach R, Grobholz R, Lammert F et al. Hepatocellular carcinoma as extracolonic manifestation of Lynch syndrome indicates SEC63 as potential target gene in hepatocarcinogenesis. Scand J Gastroenterol 2013; 48: 344–351. 23537056 10.3109/00365521.2012.752030

[bib85] Lu Z, Zhou L, Killela P, Rasheed AB, Di C, Poe WE et al. Glioblastoma proto-oncogene *SEC61γ* is required for tumor cell survival and response to endoplasmic reticulum stress. Cancer Res 2009; 69: 9105–9111. 19920201 10.1158/0008-5472.CAN-09-2775 PMC2789175

[bib86] Schuck S, Prinz WA, Thorn KS, Voss C, Walter P. Membrane expansion alleviates endoplasmic reticulum stress independently of the unfolded protein response. J Cell Biol 2009; 187: 525–536. 19948500 10.1083/jcb.200907074 PMC2779237

[bib87] Høyer-Hansen M, Jäättelä M. Connecting endoplasmic reticulum stress to autophagy by unfolded protein response and calcium. Cell Death Differ 2007; 14: 1576–1582. 17612585 10.1038/sj.cdd.4402200

[bib88] Jung V, Kindich R, Kamradt J, Jung M, Müller M, Schulz WA et al. Genomic and expression analysis of the 3q25-q26 amplification unit reveals *TLOC1/SEC62* as a probable target gene in prostate cancer. Mol Cancer Res 2006; 4: 169–176. 16547154 10.1158/1541-7786.MCR-05-0165

[bib89] Greiner M, Kreutzer B, Jung V, Grobholz R, Hasenfus A, Stöhr RF et al. Silencing of the SEC62 gene inhibits migratory and invasive potential of various tumor cells. Int J Cancer 2011; 128: 2284–2295. 20669223 10.1002/ijc.25580

[bib90] Linxweiler M, Linxweiler J, Barth M, Benedix J, Jung V, Kim YJ et al. Sec62 bridges the gap from 3q amplification to molecular cell biology in non-small cell lung cancer. Am J Pathol 2012; 180: 473–483. 22197383 10.1016/j.ajpath.2011.10.039

[bib91] Linxweiler M, Bochen F, Schick B, Wemmert S, Al Kadah B, Greiner M et al. Identification of *SEC62* as a potential marker for 3q amplification and cellular migration in dysplastic cervical lesions. BMC Cancer 2016; 16: 676. 27553742 10.1186/s12885-016-2739-6 PMC4995743

[bib92] Wemmert S, Lindner Y, Linxweiler J, Wagenpfeil S, Bohle RM, Niewald M et al. Sec62 as a new biomarker for poor prognosis in advanced head and neck squamous cell carcinoma. Oncol Lett 2016; 11: 1661–1670. 26998059 10.3892/ol.2016.4135 PMC4774472

[bib93] Weng L, Du J, Zhou Q, Cheng B, Li J, Zhang D et al. Identification of cyclin B1 and Sec62 as biomarkers for recurrence in patients with HBV-related hepatocellular carcinoma after surgical resection. Mol Cancer 2012; 11: 39. 22682366 10.1186/1476-4598-11-39 PMC3439291

[bib94] Greiner M, Kreutzer B, Lang S, Jung V, Cavalié A, Unteregger G et al. Sec62 protein level is crucial for the ER stress tolerance of prostate cancer. Prostate 2011; 71: 1074–1083. 21557272 10.1002/pros.21324

[bib95] Heselmeyer K, Macville M, Schrock E, Blegen H, Hellstrom AC, Shah K et al. Advanced-stage cervical carcinomas are defined by a recurrent pattern of chromosomal aberrations revealing high genetic instability and a consistent gain of chromosome arm 3q. Genes Chromosomes Cancer 1997; 19: 233–240. 9258658

[bib96] Allen DG, White DJ, Hutchins AM, Scurry JP, Tabrizi SN, Garland SM et al. Progressive genetic aberrations detected by comparative genomic hybridization in squamous cell cervical cancer. Br J Cancer 2000; 83: 1659–1663. 11104563 10.1054/bjoc.2000.1509 PMC2363460

[bib97] Dehan E, Ben-Dor A, Liao W, Lipson D, Frimer H, Rienstein S et al. Chromosomal aberrations and gene expression profiles in non-small cell lung cancer. Lung Cancer 2007; 56: 175–184. 17258348 10.1016/j.lungcan.2006.12.010

[bib98] Chang YC, Yeh KT, Liu TC, Chang JG. Molecular cytogenetic characterization of esophageal cancer detected by comparative genomic hybridization. J Clin Lab Anal 2010; 24: 167–174. 20486198 10.1002/jcla.20385 PMC6647568

[bib99] Haverty PM, Hon LS, Kaminker JS, Chant J, Zhang Z. High-resolution analysis of copy number alterations and associated expression changes in ovarian tumors. BMC Med Genomics 2009; 2: 21. 19419571 10.1186/1755-8794-2-21 PMC2694826

[bib100] Bockmühl U, Schwendel A, Dietel M, Petersen I. Distinct patterns of chromosomal alterations in high- and low-grade head and neck squamous cell carcinomas. Cancer Res 1996; 56: 5325–5329. 8968077

[bib101] Sheu JJ, Lee CH, Ko JY, Tsao GS, Wu CC, Fang CY et al. Chromosome 3p12.3-p14.2 and 3q26.2-q26.32 are genomic markers for prognosis of advanced nasopharyngeal carcinoma. Cancer Epidemiol Biomarkers Prev 2009; 18: 2709–2716. 19815638 10.1158/1055-9965.EPI-09-0349

[bib102] Hagerstrand D, Tong A, Schumacher SE, Ilic N, Shen RR, Cheung HW et al. Systematic interrogation of 3q26 identifies *TLOC1* and *SKIL* as cancer drivers. Cancer Discov 2013; 3: 1044–1057. 23764425 10.1158/2159-8290.CD-12-0592 PMC3770815

[bib103] Zimmermann R, Müller L, Wullich B. Protein transport into the endoplasmic reticulum: mechanisms and pathologies. Trends Mol Med 2006; 12: 567–573. 17071140 10.1016/j.molmed.2006.10.004

[bib104] Cross BCS, McKibbin C, Callan AC, Roboti P, Piacenti M, Rabu C et al. Eeyarestatin I inhibits Sec61-mediated protein translocation at the endoplasmic reticulum. J Cell Sci 2009; 122: 4393–4400. 19903691 10.1242/jcs.054494 PMC2779136

[bib105] Garrison JL, Kunkel EJ, Hedge RS, Taunton J. A substrate-specific inhibitor of protein translocation into the endoplasmic reticulum. Nature 2005; 436: 285–289. 16015336 10.1038/nature03821

[bib106] Schäuble N, Cavalié A, Zimmermann R, Jung M. Interaction of Pseudomonas aeruginosa Exotoxin A with the human Sec61 complex suppresses passive calcium efflux from the endoplasmic reticulum. Channels 2014; 8: 76–83. 24088629 10.4161/chan.26526 PMC4048345

[bib107] Baron L, Paatero AO, Morel JD, Impens F, Guenin-Macé L, Saint-Aurer S et al. Mycolactone subverts immunity by selectively blocking the Sec61 translocon. J Exp Med 2016; 213: 2885–2896. 27821549 10.1084/jem.20160662 PMC5154940

[bib108] McKenna M, Simmonds RE, High S. Mechanistic insights into the inhibition of Sec61-dependent co- and posttranslational translocation by mycolactone. J Cell Sci 2016; 129: 1404–1415. 26869228 10.1242/jcs.182352 PMC4852723

[bib109] Paatero AO, Kellosalo J, Dunyak BM, Almaliti J, Gestwicki JE, Gerwick WH et al. Apratoxin Kills Cells by Direct Blockade of the Sec61 Protein Translocation Channel. Cell Chem Biol 2016; 23: 561–566. 27203376 10.1016/j.chembiol.2016.04.008

[bib110] Carpenter Jr WT, Davis JM. Another view of the history of antipsychotic drug discovery and development. Mol Psychiatry 2012; 17: 1168–1173. 22889923 10.1038/mp.2012.121

[bib111] Zacharski LR, Moritz TE, Haakenson CM, O’Donnell JF, Ballard HS, Johnson GJ et al. Chronic calcium antagonist use in carcinoma of the lung and colon: a retrospective cohort observational study. Cancer Invest 1990; 8: 451–458. 2176124 10.3109/07357909009012067

[bib112] Denmeade SR, Jakobsen CM, Janssen S, Khan SR, Garrett ES, Lilja H et al. Prostate-specific antigen-activated thapsigargin prodrug as targeted therapy for prostate cancer. J Natl Cancer Inst 2003; 95: 990–1000. 12837835 10.1093/jnci/95.13.990

[bib113] Christensen SB, Skytte DM, Denmeade SR, Dionne C, Møller JV, Nissen P et al. A Trojan horse in drug development: targeting of thapsigargins towards prostate cancer cells. Anticancer Agents Med Chem 2009; 9: 276–294. 19275521 10.2174/1871520610909030276

[bib114] Denmeade SR, Mhaka AM, Rosen DM, Brennen WN, Dalrymple S, Dach I et al. Engineering a prostate-specific membrane antigene-activated tumor endothelial cell prodrug for cancer therapy. Sci Transl Med 2012; 4: 140ra86. 10.1126/scitranslmed.3003886 PMC3715055 22745436

[bib115] Mahalingam D, Wilding G, Denmeade S, Sarantopoulas J, Cosgrove J, Cetnar J et al. Mipsagargin, a novel thapsigargin-based PSMA-activated prodrug: results of a first-in-man phase I clinical trial in patients with refractory, advanced or metastatic solid tumors. Br J Cancer 2016; 114: 986–994. 27115568 10.1038/bjc.2016.72 PMC4984914

